# Genomic epidemiology of a Bacillus cereus bacteraemia outbreak linked to contaminated hospital laundry

**DOI:** 10.1099/mgen.0.001487

**Published:** 2025-09-04

**Authors:** Serena Bosica, Anna Janowicz, Teresa Romualdi, Mattia Ferrara, Marco Di Domenico, Roberta Di Romualdo, Giovanna Alessia Robbe, Violeta Di Marzio, Lisa Di Marcantonio, Silvia Di Zacomo, Chiara Di Iorio, Andrea Stanziale, Giuliano Garofolo, Francesco Pomilio, Paolo Fazii

**Affiliations:** 1 Istituto Zooprofilattico Sperimentale dell’Abruzzo e del Molise ‘G. Caporale’, Campo Boario 64100 Teramo, Italy; 2 Clinical Microbiology and Virology Unit, ‘Spirito Santo’ Hospital, Via Fonte Romana n. 8, 65124 Pescara, Italy

**Keywords:** *Bacillus cereus*, bloodstream infection, genomics, linens, outbreak

## Abstract

This study investigated an increase in *Bacillus cereus* bacteraemia cases amongst hospitalized patients in Italy during the summer of 2023. To precisely characterize the outbreak, we performed extensive genomic investigation, including both short- and long-read sequencing technologies, combined with bioinformatics analysis. This genomic approach enabled us to identify the putative source of the outbreak and understand the transmission dynamics of this opportunistic pathogen within the hospital. Our investigation revealed a complex, polyclonal contamination pattern traced to contaminated hospital laundry. Whole-genome sequencing (WGS) analysis identified multiple *B. cereus* sequence types (STs) in both clinical and environmental samples, with three predominant STs (ST-2184, ST-163 and ST-365) found in both. These STs, previously implicated in bloodstream infection (BSI) outbreaks, raise concerns about their potential as healthcare-associated pathogens. SNP-based phylogenetic analysis provided strong evidence linking human and environmental isolates, with close genetic relatedness observed between isolates from patients and those from laundered scrubs, transport trucks and bed linens. The study highlights the potential for laundry-mediated pathogen transmission in healthcare settings and underscores the importance of stringent laundry hygiene practices. Improved laundry procedures successfully resolved the *B. cereus* bacteraemia outbreak. This study demonstrates the power of WGS-based methodologies for investigating and resolving outbreaks, informing targeted infection control measures and ultimately enhancing patient safety.

**Impact Statement**

This study demonstrates the power of comprehensive genomic analysis to investigate and resolve a complex polyclonal *Bacillus cereus* bacteraemia outbreak in Italian hospitals. By integrating short- and long-read whole-genome sequencing (WGS) with bioinformatics tools, including core-genome MLST (cgMLST) and SNP-based phylogenetic analyses, we precisely characterized the outbreak’s genetic diversity and transmission dynamics. Our findings revealed contaminated hospital laundry as the source of the outbreak, underscoring a significant yet often overlooked route of pathogen transmission in healthcare settings. The identification of specific *B. cereus* sequence types, some with prior links to bloodstream outbreaks, raises concerns about their potential role in healthcare-associated infections. This investigation demonstrates the utility of WGS-based methodologies and advanced clustering methods for informing targeted infection control measures, ultimately enhancing patient safety and emphasizing the importance of stringent laundry hygiene practices in hospitals.

## Data Summary

The core-genome MLST (cgMLST) and accessory genome schemas generated for this study are available in the Zenodo repository under DOI: https://doi.org/10.5281/zenodo.15041223. This dataset includes the target sequences and report from the cgMLST Target Definer.

The raw reads for 89 strains of *Bacillus cereus* were deposited in NCBI under BioProject PRJNA1226552, and their specific accession numbers are listed in Table S1.

Additionally, the three *B. cereus* genomes used as references for SNP analysis were deposited in the NCBI database under the same BioProject PRJNA1226552 with BioSample accession numbers SAMN46999438, SAMN46999439 and SAMN46999440.

## Introduction

*Bacillus cereus*, a Gram-positive, spore-forming bacterium, is ubiquitously found in soil, water, air and food [1]. Its heat-resistant spores enable survival in extreme environments and potential transmission through the food chain. Although primarily associated with food poisoning due to its enterotoxin production [2,3], *B. cereus* has an important role as a causative agent of healthcare-acquired bloodstream infections (BSIs), in particular in neonates, immunocompromised individuals and critically ill patients [4].

The clinical presentation of *B. cereus* BSI is influenced by host factors and the underlying source of infection. In neonates and immunocompromised individuals, *B. cereus* BSI frequently manifests as a fulminant infection, characterized by rapid onset of fever, lethargy and hypotension that may progress to septicaemia [2,4]. Moreover, *B. cereus* BSI can lead to serious complications such as meningitis, endocarditis or pneumonia, particularly in immunocompromised hosts. Conversely, immunocompetent individuals may experience a less severe or even asymptomatic course of *B. cereus* BSI. However, localized infections such as catheter-related BSIs, endophthalmitis or soft tissue infections can occur, depending on the source and route of infection [4,6]. Accurate and timely diagnosis of *B. cereus* BSI is crucial for appropriate treatment and prevention of complications. However, distinguishing true BSIs from blood culture contamination remains a challenge due to the ubiquity of strains of *B. cereus* and its spores in most environments [4]. Multiple positive blood cultures identified as *B. cereus*, supporting a characteristic clinical presentation, are essential for accurate diagnosis.

The virulence of *B. cereus* is attributed to an array of secreted factors, including pore-forming toxins, haemolysins, phospholipases and proteases [1,7, 8]. Amongst these, the nonhaemolytic enterotoxin (nhe), haemolysin BL (hbl) and cytotoxin K (cytK) are major contributors to diarrhoeal disease [8]. Additionally, the cereulide emetic toxin is responsible for the emetic syndrome associated with *B. cereus* food poisoning [9,11]. These virulence factors facilitate evasion of host immune responses, tissue invasion and subsequent cellular damage. Moreover, the bacterium’s ability to form resilient endospores allows it to withstand harsh environmental conditions, including disinfection and sterilization protocols, contributing to its persistence in healthcare settings [11].

The persistence of *B. cereus* spores in the hospital environment and its diverse transmission routes pose challenges for infection control, and *B. cereus* outbreaks have been linked to the use of pharmaceutical products and medical devices including catheters, endoscopes, reusable ventilator airflow sensors and contaminated alcohol prep pads. Recent outbreaks have also linked contaminated hospital laundry to *B. cereus* transmission in neonates, underscoring the importance of stringent laundry hygiene practices [12]. Furthermore, asymptomatic carriage by healthcare workers can lead to inadvertent contamination, highlighting the need for meticulous hand hygiene and appropriate personal protective equipment [13,14]. Outbreaks of *B. cereus* can, however, be effectively controlled by identifying and eliminating the contaminated items or modifying the hospital linen laundering procedures [12,15].

Whole-genome sequencing (WGS) has emerged as a powerful tool in tracking the sources of *B. cereus* infections [16,17]. By analysing the entire genome of *B. cereus* strains, WGS can identify subtle genetic variations that distinguish different isolates. This enables precise identification of clusters of related infections, even when traditional epidemiological methods fail to establish clear links. Tools such as core-genome MLST (cgMLST) and SNP analysis facilitate clustering and comparison of *B. cereus* isolates [18]. This ability to trace the spread of *B. cereus* within hospitals is crucial in identifying contaminated sources and implementing effective infection control measures to prevent further outbreaks.

This study reports an unusual increase in *B. cereus* bacteraemia cases amongst hospitalized patients during the summer of 2023. The primary objective of this investigation was to comprehensively characterize the isolated strains using WGS-based methodologies and to establish epidemiological links between human infections and potential sources of contamination within the healthcare facility.

## Methods

### Bacterial isolates and culture conditions

Between June and December 2023, a total of 152 isolates of *B. cereus* were collected from three hospitals in Southern Italy: ‘Spirito Santo’ Hospital of Pescara (*n*=137; 80 clinical, 57 environmental), ‘S. Massimo’ Hospital of Penne (*n*=7, clinical only) and ‘S.S. Trinità’ Hospital of Popoli (*n*=8, clinical only).

**Fig. 1.**
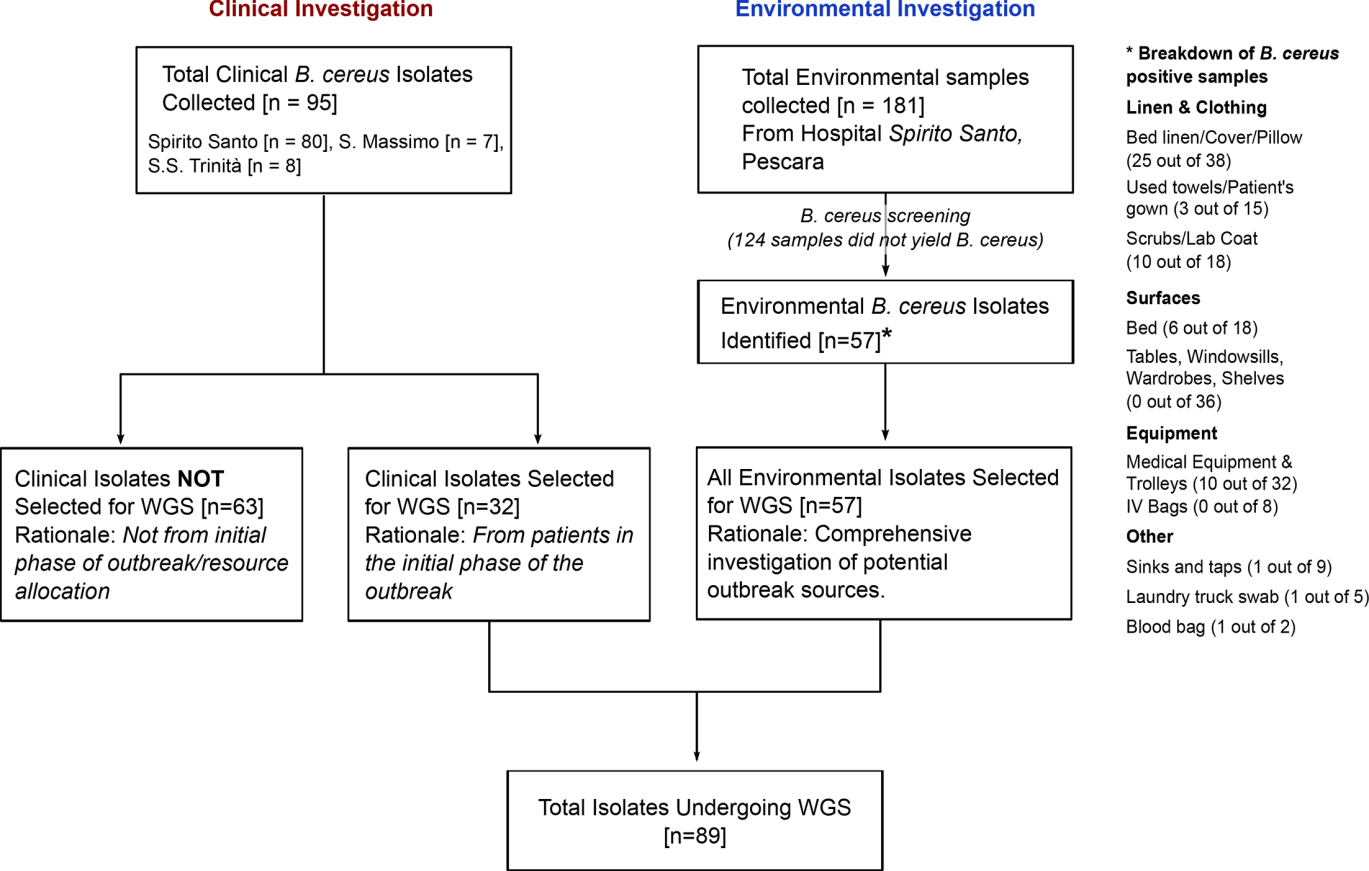
Schematic representation of the sampling process and isolate selection for WGS analysis. The flowchart details the collection of *B. cereus* isolates from both clinical cases and environmental samples, outlining the criteria used to select a total of 89 isolates for subsequent WGS. The right panel details the breakdown of *B. cereus*-positive samples from specific environmental sources (number of positive isolates per total samples).

Ninety-five isolates were recovered from clinical samples obtained from epidemiologically unrelated patients across 25 different hospital wards. The remaining 57 isolates were recovered from environmental samples collected in the ‘Spirito Santo’ Hospital of Pescara. These samples were taken from various locations within the hospital, including interested wards, a storage room for clean linens and scrubs, and comprised linens swabbed before use, one blood bag, equipment and work uniforms. A schematic diagram of the sampling process and isolate selection is shown in Fig. 1.

Clinical samples (*n*=95) consisted of blood cultures (*n*=60), urine cultures (*n*=2), wound swabs (*n*=3), respiratory samples (*n*=4), cerebrospinal fluid (*n*=1) and microbiology surveillance swabs from hospitalized patients (*n*=25). The clinical samples were cultured on Tryptic Soy Agar (TSA) with 5% sheep blood, Chocolate Agar (Vacutest Kima, Piove di Sacco, Padua, Italy), MacConkey Agar, Mannitol Salt Agar and Sabouraud Dextrose Agar (Liofilchem, Roseto degli Abruzzi, Teramo, Italy) and incubated for 48 h. The environmental swabs were inoculated onto TSA with 5% sheep blood (Vacutest Kima, Piove di Sacco, Padua, Italy) and incubated for 48 h in aerobic conditions at 37 °C ± 1 °C. Isolates were identified as *B. cereu*s using MALDI-TOF MS (bioMérieux, Lyon, France). In case of positivity for *B. cereus*, one colony was chosen randomly and seminated on a fresh TSA plate for further analysis.

### Isolate selection for genomic analysis

For WGS and subsequent genomic analyses, a subset of 89 isolates was selected. This subset comprised 32 clinical isolates from 14 different hospital wards (Table S2, available in the online Supplementary Material), primarily from the initial phase of the outbreak investigation, and 57 environmental isolates collected during targeted sampling. The selection strategy was guided by the need for timely genomic data to inform the real-time outbreak investigation and facilitate infection control measures. The selected isolates are described in Table S1, and the flowchart for the selection process is shown in Fig. 1.

### Genomic DNA extraction

*B. cereus* isolates were subcultured on Blood Agar plates (Liofilchem, Roseto degli Abruzzi, Teramo, Italy) and incubated at 37 °C ± 1 °C for 24 h. The species of the subcultured strain was re-confirmed with the MALDI Biotyper^®^ MBT^™^ smart instrument (Bruker Daltonik, Bremen, Germany) using two databases: BDAL and SR. The DNA extraction for each strain was conducted according to the method described by Portmann *et al.* [19], with minor modifications. Specifically, we used a chicken egg lysozyme solution at a concentration of 20 mg ml^−1^ (Sigma-Aldrich, Milan, Italy), followed by DNA purification using the QIAamp DNA Mini Kit (Qiagen, Hilden, Germany) according to the manufacturer’s instructions. The quality of the DNA was evaluated by measuring the absorbance (A) using a BioSpectrometer fluorescence (Eppendorf, Milan, Italy), specifically focusing on the A260/280 and A260/230 ratios.

### PCR amplification of Ba813

Genomic DNA was used to detect a fragment of Ba813, as previously described [20]. Briefly, the Ba813 marker was amplified with primers Ba813-R1 (5′-TTAATTCACTTGCAACTGATGGG-3′) and Ba813-R2 (5′-AACGATAGCTCCTACATTTGGAG-3′). Each 50 µl PCR reaction mixture contained 400 µM of each dNTP, 50 pmol of each primer, 50 U Taq DNA polymerase and 30–50 ng of template DNA. The PCR cycling conditions were as follows: an initial denaturation at 94 °C for 5 min, followed by 30 cycles of 94 °C for 1 s, 59 °C for 1 min and 72 °C for 1 min, with a final extension at 72 °C for 7 min. PCR products were subsequently analysed on a QIAxcel electrophoresis system (Qiagen). DNA from *Bacillus anthracis* was used as a positive control.

### Whole-genome sequencing

Genomic DNA from a set of 89 isolates of *B. cereus* was fully sequenced. Briefly, genomic libraries were prepared using the DNA Illumina Prep (San Diego, CA) and sequenced on the Illumina NextSeq 2000 platform, employing a 300-cycle setting for 150 paired-end reads. Raw reads were deposited in NCBI under BioProject PRJNA1226552, and the accession numbers are listed in Table S1. Trimming and quality control processes were performed using Fastp v0.23.1 [21] and FastQC v0.11.5 [22], respectively. *De novo* assembly of the sequences was performed using Shovill 1.1.0 (https://github.com/tseemann/shovill), with the assembled genomes subsequently quality-checked by QUAST v4.3 [23].

A subset of three strains isolated from clinical samples belonging to ST-163, ST-365 and ST-2184 was additionally sequenced with Oxford Nanopore Technologies for hybrid assembly (short and long reads) to be used as reference genomes for SNP analysis. Sequencing was performed on the MinION instrument (Oxford Nanopore Technologies, Oxford, UK) using the Native Barcoding Kit 24 V14 and a FLO-MIN114 R10 flow cell, with basecalling in Super Accurate (SUP) mode, filtering reads >1 kb and Q-score >20. Hybrid assembly was conducted in the Genpat platform (https://github.com/genpat-it/) using a hybrid analysis pipeline with Unicycler [24], incorporating Illumina short reads and Nanopore long reads. Quality control metrics and assembled contigs were generated. The three genomes were deposited in NCBI under BioProject PRJNA1226552 with biosample accession numbers SAMN46999438, SAMN46999439 and SAMN46999440.

### *In silico* characterization of strains

The assemblies were used for *in silico* species identification using KmerFinder, available in the in-house pipeline [25]. All assembled genomes were then classified taxonomically with Btyper3 version 3.2.0 using the recently published nomenclature [26]. ABRicate v 1.0.1 (https://github.com/tseemann/abricate) in conjunction with the MEGARes database (6,635 sequences), updated on 23 February 2023, was used to detect antimicrobial resistance (AMR) genes [27]. The same tool was additionally used to identify plasmid incompatibility (Inc) groups based on sequences found in the PlasmidFinder [28] database (460 sequences). Virulence factors were detected using both BTyper3 and ABRicate with the VFDB database [29]. All analyses were performed using default settings. The MOB-recon module in Mob-suite v3.0.0 [30] was used to classify the contigs from long- and short-read hybrid assemblies as chromosomal or plasmid-derived and to identify specific plasmid features.

### MLST and cgMLST

The assemblies of 89 strains sequenced in this study were analysed using MLST and cgMLST. The analyses were performed using Ridom SeqSphere+ version 6.0.2 using default settings. MLST profiles were assigned using the schema for *B. cereus* (i.e. *glp*, *gmk*, *ilv*, *pta*, *pur*, *pyc* and *tpi*) available in PubMLST [31,32]. Two novel STs, ST-3280 and ST-3296, were assigned after submission of allele data to the PubMLST database.

An ad hoc cgMLST schema comprising 3,741 target genes was created in SeqSphere+ using the *B. anthracis* GCF_001683155.1 genome as a reference seed. For each isolate, this schema was used to determine its cgMLST profile, defined by the specific alleles identified at each of these target loci. A neighbour-joining (NJ) tree was then generated by calculating pairwise distances based on the number of allelic mismatches between these profiles, excluding loci with missing allele data from comparisons. The mid-point rooted tree was annotated using the iTOL online tool [33].

### SNP analysis

SNPs were identified using the In Silico Genotyper (ISG), v 0.16.10 [34]. We applied default filters to exclude SNPs from duplicated regions, setting the minimum quality threshold at a Phred score of 30 and the minimum allele frequency at 90% across all samples. The SNP identification utilized the ISG pipeline, incorporating BWA-MEM (version 0.712-r1039) for alignment and GATK (version 3.9) for SNP calling. SNP calling was performed based on alignments to the chromosomal DNA of the three reference strains obtained by hybrid assembly. The clean, unique variants were used to generate MST with the PHYLOViZ 2.0 online tool, using the pairwise comparison method [35].

## Results

A total of 152 isolates of the *B. cereus* group were isolated from patients and the environment during the course of the outbreak. The preliminary species identification was performed in the clinical microbiology laboratory using combined phenotypic and MALDI-TOF results, and the isolates identified as *B. cereus* were sent to a regional institute for confirmation and additional in-depth molecular analysis. A subset of 89 isolates, comprising 32 patient isolates and 57 environmental isolates, was prioritized for WGS based on criteria developed during the real-time outbreak investigation. These included 32 isolates from hospitalized patients and 57 environmental isolates (Table S1).

We further performed a PCR assay to detect the 277 bp fragment Ba813, which was previously used to discriminate between * B. cereus* and *B. anthracis* [20], but recent studies have shown its presence in strains of *B. cereus* isolated from BSIs. We detected 26 isolates positive for the Ba813 target in our set of isolates. These included both human and environmental isolates (Tables 1 and S1).

**Table 1.** Characteristics of *B. cereus* isolates assigned to different sequence types (STs)

MLST	Total isolate count	Clinical isolates	BTyper3 genomospecies	Toxin profile	PCR Ba813	cgMLST cluster (no. of strains in cluster)
**ST-8**	3	0	*B. cereus s.s*. biovar Thuringiensis	nheA/B/C-hblA/B/C/D-cytK-2-sph-bpsE-cry1	−	C10 (2)
**ST-26**	1	0	*Bacillus mosaicus* subsp. *cereus*	nheA/B/C-sph-bpsE	−	
**ST-57**	1	0	*B. mosaicus*	nheA/B/C-cytK-2-sph-bpsE/H	−	
**ST-73**	6	0	*B. cereus s.s*.	nheA/B/C-hblA/B/C/D-cytK-2-sph-bpsD/E/H	−	C4 (3); C5 (3)
**ST-163**	19	12	*B. mosaicus*	nheA/B/C-sph-bpsE	+	C2 (12); C7 (2)
**ST-167**	3	0	*B. mosaicus*	nheA/B/C-hblA/B/C/D-sph-bpsE/H	+	C6 (3)
**ST-177**	1	0	*B. cereus s.s*.	nheA/B/C-hblA/B/C/D-cytK-2-sph-bpsE/H	−	
**ST-246**	1	1	*B. mosaicus*	nheA/B/C-hblA/B/C/D-sph-bpsE/H	−	
**ST-365**	15	11	*B. mosaicus*	nheA/B/C-sph-bpsE	+	C3 (10); C8 (2); C9 (2)
**ST-999**	1	0	*B. cereus s.s*.	nheA/B/C-hblA/B/C/D-cytK-2-sph-bpsE/H	−	
**ST-1425**	2	0	*B. mosaicus*	nheA/B/C-sph-bpsE/H	−	
**ST-1465**	1	0	*B. cereus s.s*.	nheA/B/C-hblA/B/C/D-cytK-2-sph-bpsE	−	
**ST-1777**	1	0	*B. mosaicus*	nheA/B/C-hblA/B/C/D-sph-bpsE	−	
**ST-2184**	30	5	*B. mosaicus*	nheA/B/C-sph-bpsE/H	−	C1 (30)
**ST-3280**	2	1	*B. mosaicus*	nheA/B/C-sph-bpsE	+	
**ST-3296**	2	2	*B. mosaicus*	nheA/B/C-sph-bpsE/H	+	

MLST analysis grouped the strains into 16 STs, including two novel STs (ST-3280 and ST-3296). Only six out of 16 STs contained strains isolated from the patients. Three STs, ST-163, ST-365 and ST-2184, were assigned to the largest number of strains altogether (64% of all strains); however, we observed differences between the composition of these STs. In particular, ST-2184, which was the most common ST, contained only five strains isolated from humans (17%) and 25 strains from environmental swabs (Table 1, Fig. S1). ST-163 and ST-365 instead contained more isolates obtained from humans than from the sampled environmental sources (63% and 73% of human isolates, respectively). Interestingly, these two STs were also positive for Ba813. The other three STs assigned to four clinical isolates of the *B. cereus* group were ST-246, ST-3280 and ST-3296.

*In silico* analysis using BTyper3 divided the strains into two genomospecies according to the nomenclature proposed by Carroll *et al.* [36]: *B. cereus s.s*. and *B. mosaicus* (Table 1). Additionally, one strain of *B. mosaicus* was classified as subspecies *cereus*, even though the gene coding for cereulide was not detected in its assembled genome. Most of the strains, including all those isolated from patients, belonged to *B. mosaicus* genomospecies. While we detected differences between different STs within the *B. mosaicus* genomospecies related to the number of genes coding for specific toxins, the same toxin profiles were found both in human and environmental isolates. Interestingly, while MALDI-TOF identified all the strains as *B. cereus*, KmerFinder classified seven strains as *Bacillus thuringiensis*. Within these seven strains, BTyper3 assigned three strains to *B. cereus s.s*. biovar Thuringiensis genomospecies, three to *B. mosaicus* and one to *B. cereus s.s.* (Table S1). One of these strains was isolated from a patient.

**Fig. 2.**
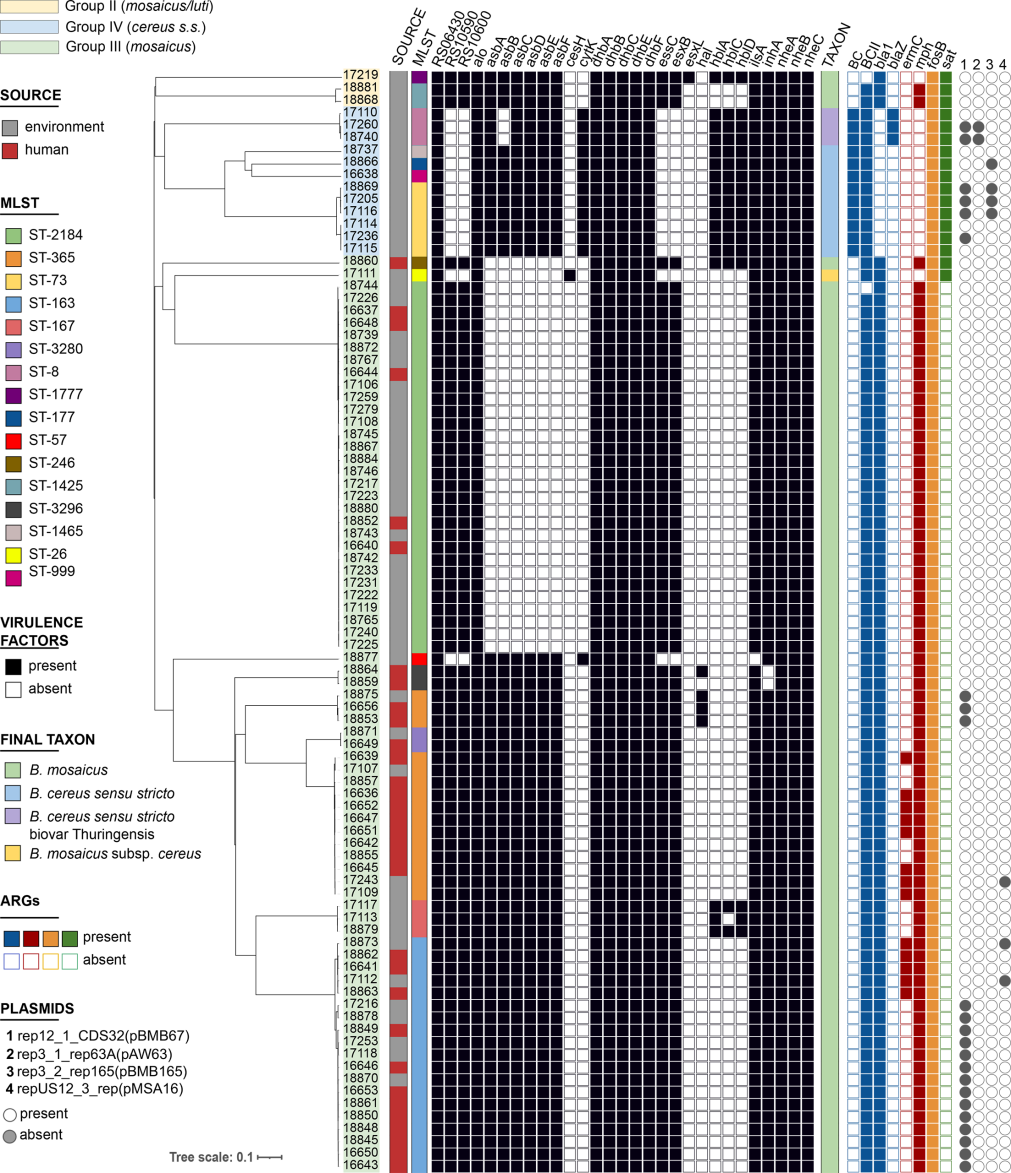
NJ tree of *B. cereus* strains isolated from human and environmental samples. A total of 89 isolates were analysed using a cgMLST schema containing 3,741 target genes. The tree was generated by pairwise comparison of the target genes with missing values ignored. Strain IDs were highlighted in yellow, blue or green according to their panC group. The IDs of the isolates were trimmed for legibility.

Analysis with cgMLST confirmed the high level of genetic diversity within our dataset, with the maximum pairwise distance detected of 3,378 alleles. The dendrogram generated based on the cgMLST profiles was congruent with PanC classification (Fig. 2). We observed three main clades that were further divided into subclades and smaller clusters of genetically similar strains. Importantly, the most populated clade corresponding to *B. mosaicus* genomospecies, or panC group III, was divided into two large subclades composed of strains isolated from both environmental and clinical samples. The first clade was mainly composed of strains assigned to ST-2184, and two strains belonging to ST-248 and ST-26, that were genetically diverse from the ST-2184 cluster. Only 6 out of 32 strains (19%) in this clade were isolated from humans. The second clade was formed by strains belonging to six different STs, with ST-163 and ST-365 represented by the highest number of strains. Moreover, 26 out of 41 strains (63 %) in this clade were isolated from humans.

Analysis of virulence factor profiles showed several differences between strains belonging to the three panC groups. In particular, in the genomes of panC group IV (*B. cereus s.s*.), we did not detect RS10590, RS10600, *ess*C and *esx*B genes coding for type VII secretion system proteins. Instead, they contained genes coding for pore-forming cytotoxin Hbl (*hbl*A, *hbl*C and *hbl*D) which, with rare exceptions, were not present in the strains from the other panC groups. The strains in panC groups II and III had similar virulence profiles, with a notable exception of strains from the subclade of panC group III belonging to ST-2184, ST-26 and ST-248 that did not contain the *asb* gene cluster involved in the biosynthesis of the siderophore petrobactin. We did not detect differences in the presence of specific virulence factors between strains isolated from environmental and human samples; rather, the differences observed were related to the phylogenetics of the strains. Similarly, we observed no differences in the AMR factors detected in strains isolated from human and environmental samples. In all analysed genomes, we identified genes conferring resistance to beta-lactams (including penicillins and cephalosporins), macrolides and fosfomycin. Furthermore, 13 strains harboured the *erm*C gene, which confers resistance to macrolides and is inducible by erythromycin. Notably, the presence of these AMR genes did not correlate with any mobile elements identified using PlasmidFinder.

To understand whether the strains isolated from patients could be genetically linked to environmental strains, we assigned cgMLST clusters using a stringent cluster inclusion threshold of six allele differences. We identified ten clusters, most of which contained only two or three strains. The three most populated clusters, C1, C2 and C3, corresponded to strains assigned to ST-2184, ST-163 and ST-365, respectively. Cluster C1 included all the strains in ST-2184, and the maximum pairwise distance between two strains in this cluster was 12 alleles. Although only five strains from C1 were isolated from humans, these strains were distant by a minimum of one allele and a maximum of four alleles from the closest environmental strains. Cluster C2 was composed mainly of human strains, and the only three environmental strains within the cluster were placed at a distance between four and six alleles from the closest strains isolated from patients. Similarly, C3 contained mainly strains obtained from human samples, but these were different from the environmental strains by two alleles only. In general, using the threshold of 6 alleles, we were able to genetically link 23 out of 32 *B. cereus* isolated from patients to at least 1 strain isolated from environmental sources. Increasing the cluster inclusion threshold to 10 core genes increased this number to 28.

To enhance the typing resolution, we conducted SNP-based cluster analysis using the hybrid genomes of three strains isolated from clinical samples belonging to the predominant STs in our dataset, ST-163, ST-365 and ST-2184, as reference strains. These strains were sequenced using both Illumina and nanopore technology, yielding a single contig of ~5.3 Mbp for each genome, corresponding to the bacterial chromosome. Additionally, each genome harboured large plasmids and several smaller mobile genetic elements, as detailed in Table S3.

**Fig. 3.**
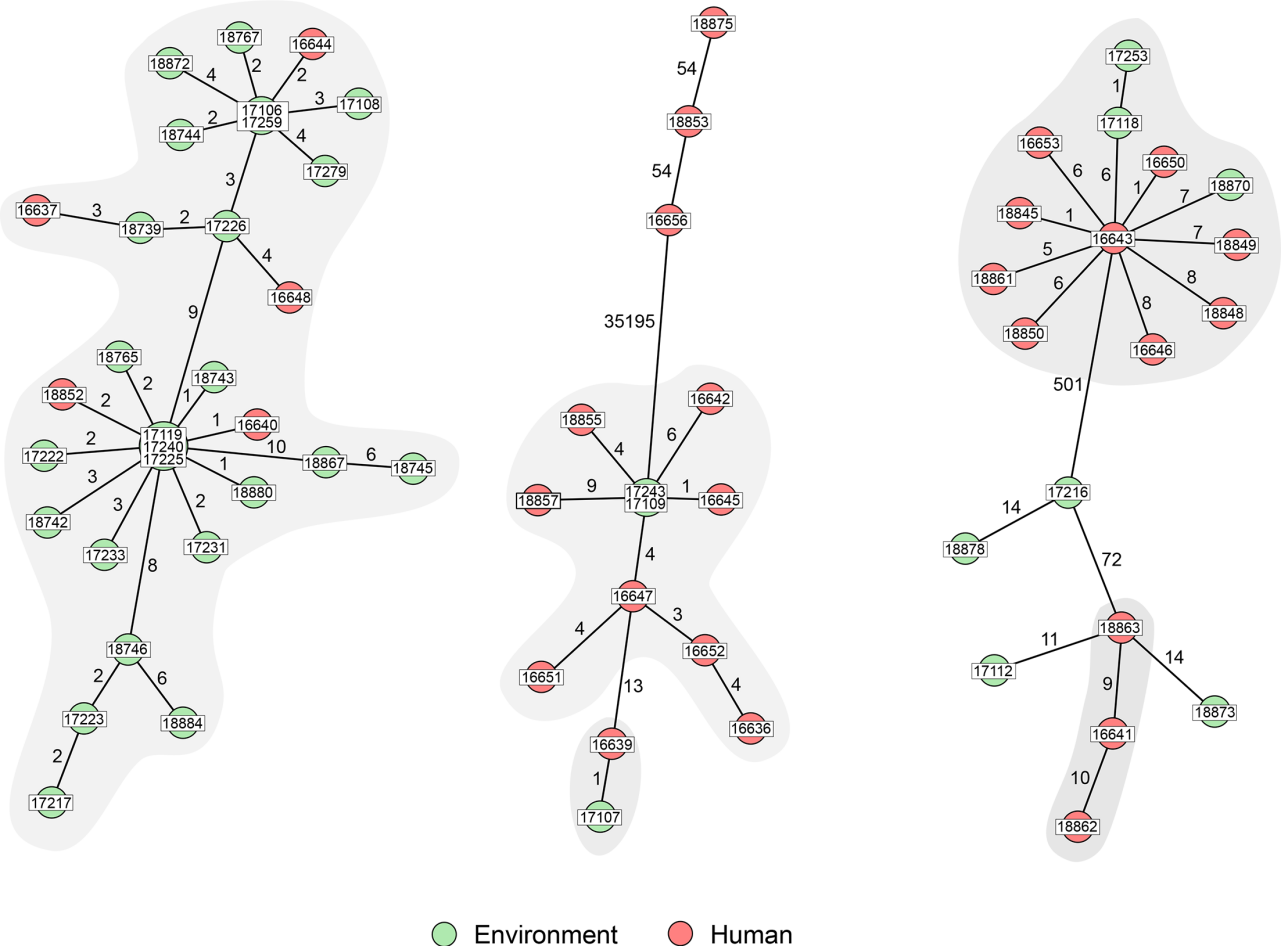
Minimum spanning trees (MSTs) generated for *B. cereus* ST-2184, ST-365 and ST-163. MST was calculated based on the identified SNP using representative strains of each ST as a reference. The distance labels correspond to the number of discriminating SNPs between neighbouring genotypes. The red colour nodes correspond to human isolates and the green nodes to environmental isolates. The IDs of the strains were trimmed for legibility.

The clustering obtained through SNP analysis showed a high degree of similarity to the results derived from cgMLST analysis (Fig. 3). However, as anticipated, the genetic distances between individual genomes increased for several strains. To maintain consistency with the clusters identified using a cgMLST cut-off of six alleles, we adjusted the threshold to ten SNPs for inclusion in SNP-based clustering. With this threshold, the maximum pairwise distances within the three principal clusters, C1, C2 and C3, increased to 16, 23 and 14 SNPs, respectively. Importantly, the minimum distances between strains isolated from humans and environmental sources were comparable to those observed in the cgMLST analysis. These distances ranged from one to four for C1, were between six and seven for C3 and equalled one for C2, as shown in Table 2. The environmental sources that were genetically closest to the human strains included freshly laundered scrubs, surface swabs from the laundry transport truck and hospital bed linens (Table S4). This evidence strongly suggests that the laundry service was likely the source of contamination within the hospital environment, subsequently affecting hospitalized patients.

**Table 2.** Features of the main genetic clusters of *B. cereus* generated using cgMLST and SNP analysis

Cluster	Strain count		Maximum pairwise distance		Minimum distance (human to environmental strain)	
	Total	Human	cgMLST	SNP typing	cgMLST	SNP typing
**C1**	30	5	12	16	1–4	1–4
**C2**	12	9	10	14	2	1
**C3**	10	8	17	23	4–7	6–7

## Discussion

*B. cereus* BSIs pose a significant threat in healthcare settings, especially amongst vulnerable patient populations including immunocompromised individuals, neonates and the critically ill, with reported mortality rates generally ranging between 20% and 40% [37,38]. The capacity of *B. cereus* to form resilient endospores contributes to its persistence in hospital environments, complicating infection control efforts and often making it challenging to definitively distinguish true BSIs from contamination or to ascertain the precise source of acquisition. Hospitalized patient populations are often inherently susceptible to opportunistic infections due to underlying conditions and frailty, particularly those recovered in units such as intensive care, neonatology, haematology and geriatrics, from which several isolates in this study originated.

In this context, our study investigated an unusual surge in *B. cereus* BSIs during the summer of 2023 amongst patients in multiple units across three hospitals. Although differentiating healthcare-associated *B. cereus* BSIs from potentially coincidental community-acquired infections in hospitalized individuals can be complex for individual cases, the finding of genomically related strains shared between patients and the hospital environment robustly supports the healthcare-associated nature of the cases within this outbreak cluster. Utilizing extensive genomic investigation, this study revealed a complex, polyclonal contamination pattern ultimately traced to contaminated hospital laundry, providing crucial insights into the transmission dynamics of this opportunistic pathogen within the healthcare setting.

WGS revealed multipl*e B. cere*us STs in both clinical and environmental samples, indicating a diverse population. However, three STs, ST-2184, ST-163 and ST-365, were predominant, representing the majority of isolates, and, importantly, were present in both clinical and environmental samples. All three of these STs have been previously implicated in BSI outbreaks [39,41], raising concerns about their potential as emerging nosocomial pathogens. Specifically, ST-365 and ST-2184 have been associated with BSIs in neonates in Taiwan [41]. While ST-2184 was isolated more frequently from the environment in our study, ST-163 and ST-365 were more prevalent amongst clinical isolates. This distribution could suggest a potentially greater pathogenic potential or transmissibility of ST-163 and ST-365 within the context of this specific outbreak.

Our analysis comparing AMR and virulence factors between clinical and environmental isolates was based on the presence of known genes as determined by WGS. Gene expression levels were not assessed in this study. Although our study did not detect differences in the presence of known virulence factors between these and other environmentally restricted STs, this does not exclude differences in virulence factor expression or the presence of previously uncharacterized virulence factors.

Our study showed some discrepancies in strain identification using different methods. MALDI-TOF consistently identified all isolates as *B. cereus*, while KmerFinder classified seven as *B. thuringiensis*. MALDI-TOF’s limited discriminatory power within the *B. cereus sensu lato* group, due to high ribosomal protein similarity, is a known challenge [42,43]. BTyper3 further resolved these seven strains into *B. cereus s.s*. biovar Thuringiensis, *B. mosaicus* and *B. cereus* s.s., highlighting the complexity of genomic-based classification in this group. BTyper3 utilizes a comprehensive database of virulence and housekeeping genes, providing a more refined classification than other genomics-based methods [26]. Accurate MALDI-TOF differentiation, especially for the *B. cereus sensu lato* group and in particular for differentiation of *B. anthracis* from less virulent species of the group, requires specific databases that may not often be routinely used in clinical laboratories. Given these observed inconsistencies and the taxonomic complexity of the *B. cereus* group, our findings highlight the importance of employing robust, genomics-based classification frameworks, such as BTyper3, in future surveillance and epidemiological studies to provide more accurate and refined taxonomic assignments compared to methods with limited discriminatory power within this group.

The presence of the *B. anthracis* marker Ba813 in ST-163 and ST-365 is noteworthy. While its role in *B. cereus* remains unclear, it could be associated with genes involved in sporulation, survival or virulence. Aoyagi *et al.* [44] demonstrated that Ba813 is not a reliable specific marker for *B. anthracis* and that Ba813-positive *B. cereus* strains are a significant cause of nosocomial BSIs in Japan [44]. This observation aligns with our findings of Ba813 presence in clinically prevalent STs, further diminishing the marker’s utility for distinguishing *B. anthracis* from *B. cereus* in clinical contexts. While the marker reliably identifies a specific *B. cereus* lineage (Cereus III) closely related to *B. anthracis* and has been frequently observed in healthcare settings, the concept of Ba813 as a direct pathogenicity or virulence marker remains understudied. Its association with clinically relevant strains is currently based more on epidemiological observation than on definitive evidence linking its presence to increased disease severity or enhanced virulence factor profiles. Thus, Ba813 serves as an important marker for tracking this lineage in outbreaks but should not be solely interpreted as a predictor of clinical outcome without further functional investigation. Future research should focus on elucidating the function of Ba813 and determining whether it directly contributes to pathogenicity or is a linked marker. This is particularly important given the clinical prevalence of the Ba813-positive STs.

SNP-based phylogenetic analysis provided strong evidence linking human and environmental isolates. The close genetic relatedness between isolates from patients and those from laundered scrubs, transport trucks and bed linens strongly implicated the hospital laundry system as the source of the outbreak. This highlights a critical, and frequently overlooked, gap in infection control: the potential for laundry-mediated pathogen transmission that may not always be investigated [37]. Moreover, while internal handling procedures aim to contain soiled linen, the presence of *B. cereus* on the transport trucks highlights this phase as a persistent potential source for cross-contamination. Previous studies, primarily from Asia [12,45, 46], have reported similar associations. Indeed, hospital textiles provide a particularly suitable environment for *B. cereus* persistence and transmission, owing to the spores' inherent resilience to standard laundering processes and the protective, fibrous nature of the textiles themselves, which can harbour spores and retain moisture conducive to germination and subsequent vegetative growth [47]. The confluence of the summer season, with high temperatures promoting *B. cereus* sporulation and growth [48], and potential shortcomings in laundry disinfection practices likely contributed to the polyclonal nature of this outbreak. *B. cereus* is environmentally ubiquitous, and therefore, numerous distinct strains were likely introduced into the laundry system. The elevated temperatures during the summer months would not selectively favour a single strain but rather could promote the sporulation and growth of various *B. cereus* strains present. Similarly, any shortcomings in laundry disinfection might reduce overall bacterial loads but could be insufficient to eradicate all strains, particularly highly resistant spores from diverse genetic backgrounds. Instead of selecting for a single dominant strain, such conditions would permit the survival and proliferation of a mixed population of *B. cereus* STs already present within the laundry environment or on incoming contaminated linens, leading to the observed polyclonal pattern in both environmental and clinical isolates. Following the implementation of improved laundry procedures, the *B. cereus* bacteraemia outbreak was effectively contained and resolved within 2 months, as evidenced by the cessation of new cases, strongly implicating the contaminated laundry as the source. Continued efforts should prioritize enhanced surveillance, rigorous adherence to infection control protocols and prompt, thorough investigation of any future outbreaks.

The polyclonal nature of the initial outbreak phase underscores a key limitation of WGS in early outbreak investigations [49]. Limited initial samples may obscure genetic connections due to the inherent heterogeneity of a contaminating population, as observed with *B. cereus*. This can lead to an underestimation of isolate relatedness, potentially delaying source identification. Indeed, this outbreak was characterized by an initial contamination source composed of multiple STs of *B. cereus*. Our WGS strategy was further shaped by the real-time nature of the investigation. An iterative approach to sequencing was adopted, initially focusing on a subset of clinical isolates to assess clonality, followed by an expanded set including targeted environmental samples guided by the infection control team. This approach, while resource-conscious and aimed at providing rapid, actionable outputs, meant that not all 152 isolates collected over the entire outbreak period were sequenced. Furthermore, some of the earliest isolates, retrospectively identified as part of the June increase in cases, were unavailable for WGS due to routine laboratory disposal protocols enacted before the outbreak was fully recognized. Consequently, while our analysis of 89 isolates provided strong evidence implicating the laundry system, the full genetic diversity and the earliest transmission dynamics might have been incompletely captured.

Furthermore, high-resolution clustering analysis revealed substantial within-cluster variation amongst the isolates (measured by pairwise distance between two most distant genomes in the cluster), even in those groups considered highly related by cgMLST and SNP analysis. This micro-diversity likely reflects the dynamic nature of *B. cereus* genomes and the presence of a heterogeneous environmental reservoir of *B. cereus*, including both genomically related and unrelated strains. Therefore, WGS data, especially when generated progressively during an active outbreak with evolving understanding and sample availability, must be interpreted cautiously, considering the outbreak timeline, sample representativeness and the practical constraints of the investigation.

Despite this limitation, WGS remains an invaluable tool for outbreak investigations, particularly when combined with high-resolution typing methods like cgMLST and SNP analysis [50]. In this case, WGS enabled us to trace seemingly disparate isolates back to the laundry service, demonstrating the power of integrating detailed genetic analysis with epidemiological data for effective outbreak control and prevention.

## Supplementary material

10.1099/mgen.0.001487 Uncited Fig. S1.

10.1099/mgen.0.001487 Uncited Supplementary Material 1.

## Abbreviations

AMR: antimicrobial resistance
BSIs: bloodstream infections
cgMLST: core-genome MLST
cytK: cytotoxin K
hbl: haemolysin BL
ISG: In Silico Genotyper
MSTs: minimum spanning trees
nhe: nonhaemolytic enterotoxin
NJ: neighbour-joining
STs: sequence types
TSA: Tryptic Soy Agar
WGS: whole-genome sequencing
