# Supplementary material for: Genomic epidemiology of a Bacillus cereus bacteraemia outbreak linked to contaminated hospital laundry
Source: Microb Genom. 2025 Sep 4;11(9):001487. doi: 10.1099/mgen.0.001487 (PMC12421256; doi:10.1099/mgen.0.001487)
Supplement: Uncited Fig. S1. [file mgen-11-01487-s001.pdf]

ST	Human Clinical	Laundered Scrubs/Lab coat	Bed Linens	Bed/mattress	Towels/gown	Blood bag	Laundry truck	Other Environmental
ST-8	0	0	0	1	0	0	0	2
ST-26	0	0	0	0	0	0	0	1
ST-57	0	0	1	0	0	0	0	0
ST-73	0	1	5	0	0	0	0	0
ST-163	12	5	0	1	0	0	1	0
ST-167	0	1	1	0	1	0	0	0
ST-177	0	0	1	0	0	0	0	0
ST-246	1	0	0	0	0	0	0	0
ST-365	11	0	3	1	0	0	0	0
ST-999	0	0	0	0	0	1	0	0
ST-1425	0	0	1	0	1	0	0	0
ST-1465	0	0	0	0	0	0	0	1
ST-1777	0	0	1	0	0	0	0	0
ST-2184	5	2	12	4	0	0	0	7
ST-3280	1	0	0	0	1	0	0	0
ST-3296	2	0	0	0	0	0	0	0

Supplementary Figure 1. Heatmap of environmental isolate distribution by source across identified STs, with human isolate counts highlighted in grey.
